# Free-surface molecular command systems for photoalignment of liquid crystalline materials

**DOI:** 10.1038/ncomms4320

**Published:** 2014-02-18

**Authors:** Kei Fukuhara, Shusaku Nagano, Mitsuo Hara, Takahiro Seki

**Affiliations:** 1 Department of Molecular Design and Engineering, Graduate School of Engineering, Nagoya University, Furo-cho, Chikusa, Nagoya 464-8603, Japan; 2 Nagoya University Venture Business Laboratory, Nagoya University, Furo-cho, Chikusa, Nagoya 464-8603, Japan

## Abstract

The orientation of liquid crystal molecules is very sensitive towards contacting surfaces, and this phenomenon is critical during the fabrication of liquid crystal display panels, as well as optical and memory devices. To date, research has focused on designing and modifying solid surfaces. Here we report an approach to control the orientation of liquid crystals from the free (air) surface side: a skin layer at the free surface was prepared using a non-photoresponsive liquid crystalline polymer film by surface segregation or inkjet printing an azobenzene-containing liquid crystalline block copolymer. Both planar-planar and homoeotropic-planar mode patterns were readily generated. This strategy is applicable to various substrate systems, including inorganic substrates and flexible polymer films. These versatile processes require no modification of the substrate surface and are therefore expected to provide new opportunities for the fabrication of optical and mechanical devices based on liquid crystal alignment.

## Abstract (web summary)

Molecular alignment in liquid crystals is conventionally achieved by surface mediation from the substrate side. Fukuhara *et al.* develop a method to photocontrol the orientation of liquid crystalline polymer films from the air side, which is guided by a photoresponsive skin layer.

## Graphical abstract

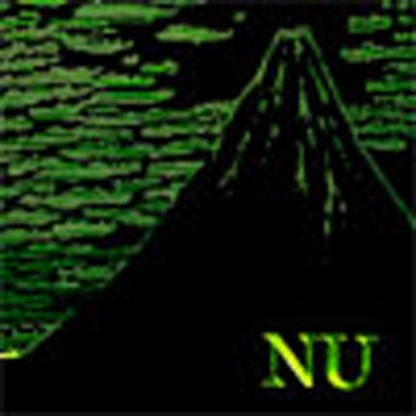

The orientation of liquid crystals (LCs) on solid substrate surfaces have been known since the work by Mauguin^1^ at the dawn of LC research. Later, Chatelin^2^ clearly revealed the rubbing effect on a substrate regarding LC orientation. Now, rubbing processes on polymer film surfaces are standard procedures used for aligning the surfaces of LCs during LC display panel and optical modulation device fabrication.

An alternative procedure to control LC orientation is to use photoreactions occurring at the substrate surface. The first demonstration of surface photoalignment control was reported by Ichimura *et al.*^3^ in 1988. The photoisomerization of an azobenzene monolayer on the surface could switch the nematic LCs that are micrometres thick between the homoeotropic and planar modes (Fig. 1, left). This system is called a ‘command surface’ or a ‘command layer’^4^,^5^,^6^,^7^,^8^. Shortly after, Gibbons *et al.*^9^, Dyadyusha *et al*.^10^ and Schadt *et al.*^11^ demonstrated that angular selective excitation with linearly polarized light (LPL) on a photoreactive polymer film enables in-plane alignment control. More than 20 years after these findings, surface photoalignment technology^12^,^13^ has recently been introduced in the industrial production of LC displays^14^. Surface photoalignment is most often applied to thermotropic nematic LCs, but many types of other LC materials, including LC polymers, can be photoaligned^6^,^15^,^16^. The mechanisms of photoalignment have recently been overviewed by Yaroshchuk and Reznikov^13^. To date, all the surface alignments have been mostly achieved through an aligning effect from the solid substrate surface.

Although the importance of the free surface (air-contacting surface) in the anchoring of mesogen orientations has long been recognized theoretically^17^,^18^ and experimentally^19^,^20^, no attempt had been made to utilize the free surface to exert active orientation control over the LC. In this communication, we report the first examples of polymer LC systems commanded by a skin layer at the free surface (Fig. 1, right). This approach was inspired by our recent results showing that the surface coverage on the free surface of photoresponsive azobenzene side chain LC polymer films leads to a homoeotropic to planar orientational change in the mesogenic groups across the entire film^21^,^22^. This pre-oriented state leads to an effective in-plane photoalignment using LPL irradiation. The free surface was covered by mixing a small amount of surface-segregating polymer and successive annealing. In our previous investigations, the entire film was composed of photoresponsive (self-alignable) LC polymers. The present approach was undertaken to demonstrate photoalignment control over non-photoresponsive LC polymer films commanded only by a photoresponsive skin layer at the free surface. The present method is particularly versatile and should offer various new applications without laborious surface modifications to the substrate, and LC alignment controls can be performed by annealing, followed by LPL irradiation or general printing methods. Using an inkjet printing process on the air side surface, photoaligned fine patterning and on-demand drawings of aligned mesogens in the polymer films are available. New photoalignment methods for the LC polymer films are proposed.

## Results

### Synthesis of polymers

**Figure 2:**
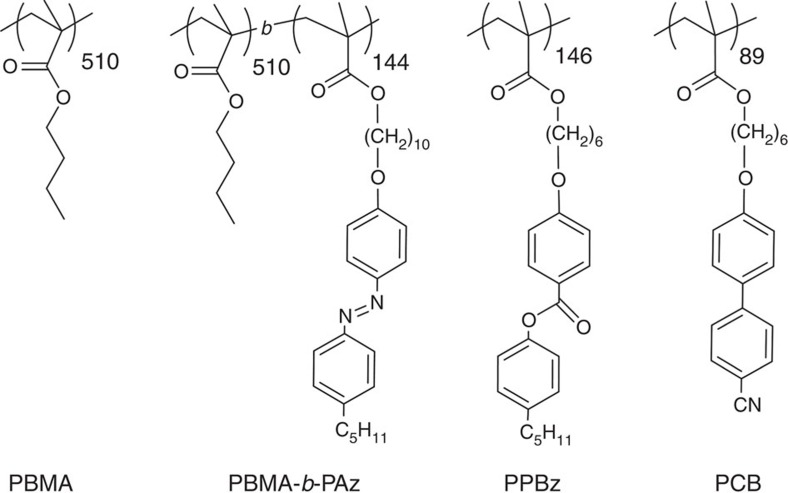
Chemical structures of polymers. PBMA is the flexible homopolymer used for the reference experiments; PBMA-*b*-PAz is the photoresponsive surface segregating the block copolymer; PPBz and PCB are the non-photoresponsive side chain homopolymers.

The synthetic procedures used to produce the monomers and polymers are described in the Supplementary Information. Figure 2 presents the chemical structures and abbreviations of the polymers employed. Poly(butyl methacrylate) (PBMA), poly(butyl methacrylate)-*block*-poly[4-(10-methacryloxydecyloxy)-4′-pentylazobenzene] (PBMA-*b*-PAz) and poly (6-cyanobiphenyloxy-1-hexyl methacrylate) (PCB) were synthesized via atom transfer radical polymerization, as previously reported^21^, and poly(4-(6-methacryloyloxyhexyloxy)-4′-pentylphenyl benzoate (PPBz) was synthesized by free radical polymerization. The number-averaged molecular masses (*M*_n_) and the polydispersity indices (*M*_w_/*M*_n_) were evaluated by gel permeation chromatography (GPC): PBMA (*M*_n_=7.2 × 10^4^, *M*_w_/*M*_n_=1.09) PBMA-*b*-PAz (*M*_n_=1.4 × 10^5^, *M*_w_/*M*_n_=1.17), PPBz (*M*_n_=6.6 × 10^4^, *M*_w_/*M*_n_=2.04) and PCB (*M*_n_=3.2 × 10^4^, *M*_w_/*M*_n_=1.11). The thermophysical properties were evaluated using differential scanning calorimetry (DSC; Supplementary Fig. 1) and polarized optical microscopy (POM): PPBz (glass-23 °C-smectic A-83 °C-isotropic); PCB (glass-50 °C-smectic A-113 °C-isotropic); PBMA-*b*-PAz (glass-57 °C-smectic C-90 °C-smectic A-118 °C-isotropic). The glass transition temperature of the PBMA homopolymer and the block copolymer was ~20 °C. PBMA-*b*-PAz was used as the photoresponsive minor blending component, and PPBz and PCB are non-photoresponsive side chain LC polymers that constitute the majority of the film. Unless stated otherwise, the LC polymer films were prepared on clean quartz plates.

### Effect of PBMA-*b*-PAz addition on the LC polymer films

**Figure 3:**
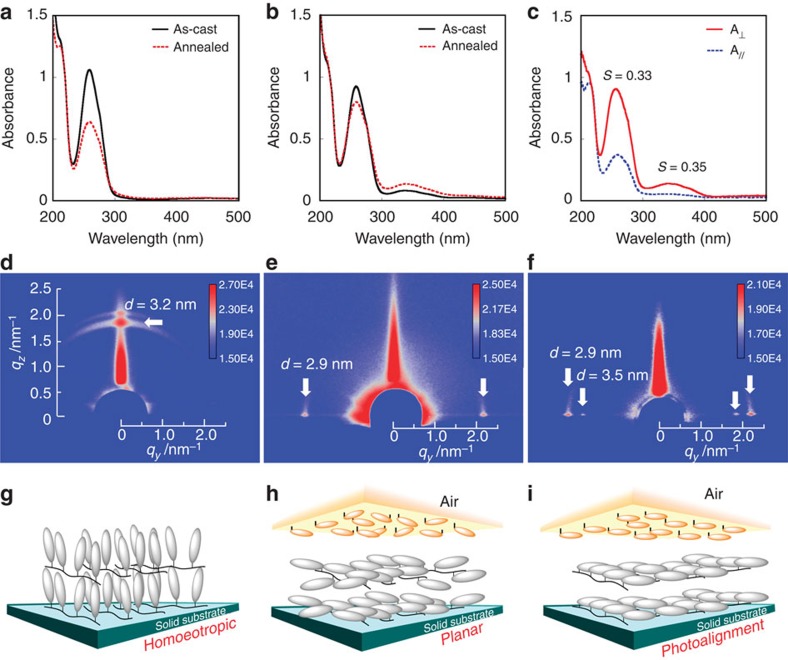
LC molecular orientations in the LC films. Ultraviolet–visible absorption spectra of a pure PPBz film (**a**) and a PBMA-*b*-PAz (3 weight %)/PPBz film (**b**). The solid and dotted lines indicate the spectra before and after annealing at 125 °C for 10 min. Polarized ultraviolet–visible absorption spectra of the annealed PBMA-*b*-PAz (3%)/PPBz film after LPL irradiation (600 mJ cm^–2^) at 80 °C taken with probing polarized light set perpendicular (A_⊥_) and parallel (A_//_) to the actinic LPL light (**c**). (**d**–**f**) the two-dimensional GI-SAXS images are shown for the above corresponding films from **a**–**c**, respectively. Schematic drawings for each state are displayed from **g** to **i**.

Figure 3 shows the ultraviolet–visible absorption spectra and the grazing incidence small angle X-ray scattering (GI-SAXS) data used to evaluate the orientations of the mesogen and SmA lamella structure.

The ultraviolet–visible absorption spectra of a pure PPBz film after annealing at 125 °C for 10 min exhibited a significant decrease in the π−π* band for phenyl benzoate at 270 nm (Fig. 3a). In the GI-SAXS data, a diffraction spot was observed in the out-of-plane direction with a *d* spacing of 3.2 nm (Fig. 3d). These data revealed that the SmA lamella and the phenyl benzoate mesogens are oriented parallel and perpendicular to the substrate, respectively. The mesogen and lamella orientations are schematically drawn in Fig. 3g. These results agree with the general understanding that rod-like mesogenic groups tend to be anchored vertically at the free surface^17^,^18^,^19^,^20^.

In contrast, when 3 weight % of PBMA-*b*-PAz was blended with the PPBz, the film exhibited only minor changes in the absorption spectrum on annealing (b), and the orientation of the lamella structure changed to the in-plane directions with *d*=2.9 nm (Fig. 3e). This orientational change should be attributed to the segregation of PBMA-*b*-PAz at the free surface during the annealing treatment, blocking the vertical anchorage of phenyl benzoate mesogens to the free surface. Before this annealing, the scattering spots in the in-plane directions with arcs were observed, indicative of disordered orientation of smectic layers (Supplementary Fig. 2). Direct evidence for the surface segregation of PBMA-*b*-PAz will be demonstrated in the next section. The molecular orientation model is displayed in Fig. 3h. We assume that this in-plane orientation can also be achieved by the molecular level orientation of PBMA-*b*-PAz thin film. When a flexible PBMA block is positioned at the top surface, the backbone of the azobenzene polymer block is preferentially oriented vertically relative to the surface. Therefore, the azobenzene mesogens should be oriented in the parallel direction, aligning the phenyl benzoate mesogens in the same direction. The absorption intensity of the π−π* (~350 nm) for azobenzene was approximately one-tenth of that for phenyl benzoate (~270 nm). As the molar extinction coefficient of the π−π* band for phenyl benzoate (~1 × 10^4^ dm^3^ mol^−1^ cm^−1^) was approximately one-third that of azobenzene^8^, the observed absorption intensity in Fig. 3b is appropriate for the sample containing 3 weight % of PBMA-*b*-PAz.

Essentially the same results were obtained with another non-photoresponsive LC polymer: PCB (Supplementary Fig. 3). In this report, data obtained with PPBz are mainly discussed because the π−π* band for phenyl benzoate is separate from that of azobenzene. This situation enables observation of the orientations held by the two mesogenic groups independently; however, the π−π* absorption band for cyanobiphenyl partially overlapped in the PCB system (Supplementary Fig. 3).

When the annealed blend polymer was irradiated using 436 nm LPL at 600 mJ cm^−2^, a strong optical in-plane anisotropy appeared (Fig. 3c). The in-plane orientational order parameter (*S*) was estimated as the following: *S*=(*A*_⊥_−*A*_||_)/(*A*_⊥_+2*A*_||_), where *A*_⊥_ and *A*_||_ denote the absorbance at the peak maximum obtained through measurements using polarized light with *E* perpendicular and parallel to that of the actinic polarized light, respectively (Supplementary Note 1). The *S* values here are determined by assuming uniaxial ordering in the in-plane direction. The photoalignment behaviour in LC polymer materials is in general more complex involving 3D ordering^23^, but, in this study, this simple evaluation is sufficient to grasp the alignment processes. The *S* values obtained for the azobenzene and phenyl benzoate bands were 0.35 and 0.33, respectively. Both order parameters agreed with one another. GI-SAXS measurements also revealed a uniformly photoaligned lamella structure in the film. The diffraction peaks became more pronounced (Fig. 3f), and additional diffraction peaks from the PBMA-*b*-PAz layer (*d*=3.5 nm) were also observed, reflecting the more ordered layer structure for both mesogens. Schematic illustrations of the mesogenic orientations in the blended films before and after LPL irradiation are shown in Fig. 3h,i. These non-photoresponsive LC mesogens are photoaligned (commanded) by the free-surface photoresponsice layer.

### Evidence for the formation of the surface skin layer

**Figure 4:**
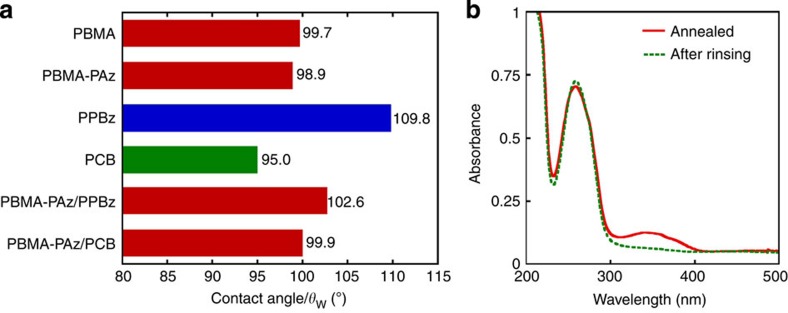
Evidence for the skin layer formation of PBMA-*b*-PAz. (**a**) Contact angle of a water droplet (*θ*_w_) on various polymer surfaces after annealing at 125 °C for 10 min. (**b**) Ultraviolet–visible absorption spectra of an annealed PBMA-*b*-PAz (3%)/PPBz film (red solid line) and the identical film after rinsing with cyclohexane at 45 °C for 5 min (green dotted line). Note that the π−π* band for the azobenzene (~350 nm) completely disappeared, while that of the phenyl benzoate (~270 nm) remained unchanged.

The contact angles of a water droplet (*θ*_w_) on various annealed polymer film surfaces are summarized in Fig. 4a. The *θ*_w_ values for the PPBz and PCB surfaces were 110° (blue bar) and 95° (green bar), respectively. The polymer films (PBMA, PBMA-*b*-PAz and PBMA-*b*-PAz (3%)/PPBz blend) usually exhibited values of ~100° (red bars). These data suggest two significant issues. First, when 3% PBMA-*b*-PAz is blended into PPBz and annealed, the surface-free energy matches that of PBMA and PBMA-*b*-PAz. Therefore, the PBMA segments of PBMA-*b*-PAz should be located at the topmost surface in the two blended films. This situation should be an entropic requirement that flexible moieties with larger mobility are segregated at the free surface^24^,^25^. Second, the surface-free energy of the pure PPBz and PCB are lower and higher than PBMA-*b*-PAz, respectively. Therefore, the surface-free energy of the LC polymer relative to PBMA-*b*-PAz was less significant. Therefore, the present method is widely applicable to many side chain LC polymers.

The existence of PBMA-*b*-PAz skin layer in the blend film was directly confirmed by transmission electron microscopy (TEM). A surface skin layer with a thickness of 20 nm was successfully observed by TEM in our previous work with a block copolymer that forms microphase separated polystyrene cylinders^21^. In that case, staining with a heavy metal compound led to a clear contrast in the microphase separated structure, confirming the existence of the skin layer. However, in the present system, no contrast would be available between the chemically similar side chain polymers (PPBz and PBMA-*b*-PAz). To validate the surface segregation of PBMA-*b*-PAz in the present system, we adopted a simple procedure using ultraviolet–visible spectroscopic measurements. The annealed blended film was immersed into an organic solvent that dissolves only PBMA-*b*-PAz. After screening various solvents, cyclohexane was used at 45 °C for 5 min. Figure 4b denotes the ultraviolet–visible absorption spectra of the annealed PBMA-*b*-PAz(3%)/PPBz film before and after rinsing with cyclohexane. After this treatment, only the π−π* absorption band at ~350 nm for azobenzene disappeared, while that of phenyl benzoate at ~270 nm remained unchanged, unequivocally indicating that PBMA-*b*-PAz is located at the outermost surface and only this block copolymer is dissolved. This result also indicates that the PBMA-*b*-PAz component almost fully migrated to the free surface.

Atomic force microscopic measurements revealed that the average film thicknesses before and after rinsing were ~150 and 130 nm, respectively, indicating that the thickness of the surface-segregated PBMA-*b*-PAz layer was ~20 nm. This thickness is comparable to that observed by TEM in our previous system^21^.

### Planar-planar mode photopatterning from the surface layer

**Figure 5:**
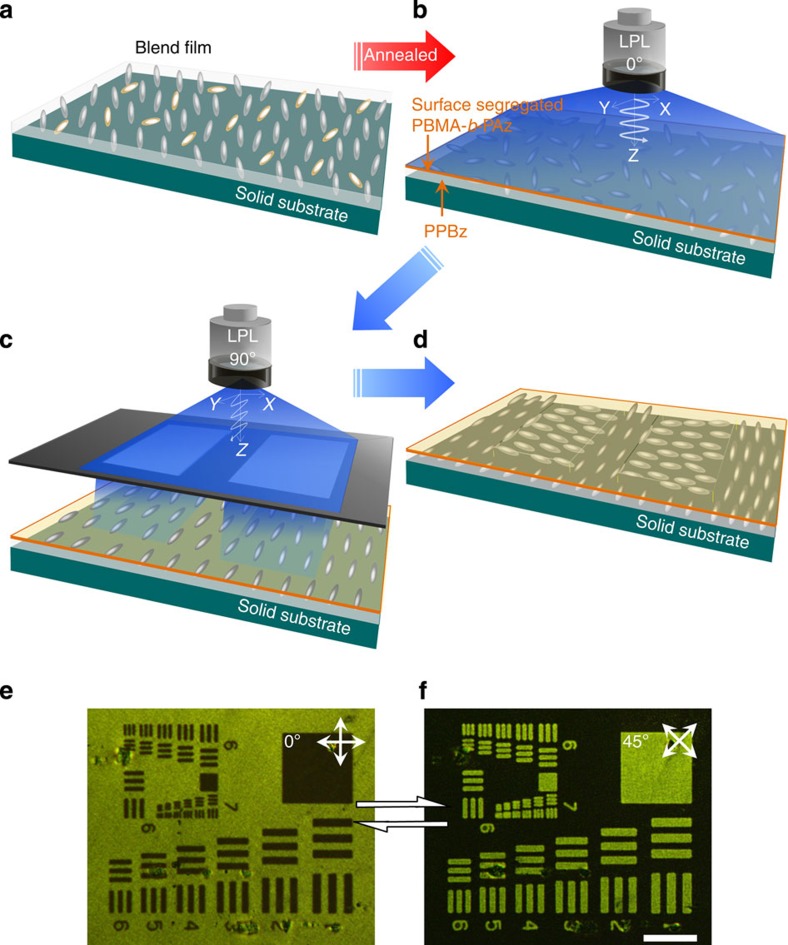
Planar-planar mode orientation patterning by LPL irradiation. Scheme showing the procedures for the in-plane photopatterning of the side chain polymer films (**a**–**c**). (**a**) An as-cast film of the side chain polymer (PPBz or PCB) blended with a small amount of PBMA-*b*-PAz (3 weight %) prepared by simple solvent casting or spincasting. (**b**) After annealing at 125 °C for 10 min, a skin layer of photoresponsive PBMA-*b*-PAz is formed at the free surface. At this stage, the side chain mesogens adopt a random planar orientation. This film is irradiated with LPL at 80 °C for 10 min (600 mJ cm^−2^) across the entire area of the film, providing a uniform, in-plane aligned film. (**c**) Successive patterning irradiation with LPL rotated 90° through a photomask, patterning in a planar-planar orientation mode for the PPBz film (scheme in **d**). (**e**,**f**) show the POM images under crossed polarizers that were rotated 45° from each other. Note that the positive and negative tone patterns are reversed. Scale bar, 100 μm.

Next, we attempted to develop patterns with the in-plane LC orientations triggered via the surface skin layer. Figure 5 shows a scheme with the procedures and resulting patterns. A cast PBMA-*b*-PAz (3%)/PPBz blend film (~5 μm thick) was prepared (Fig. 5a) and annealed at 125 °C for 10 min to form a PBMA-*b*-PAz skin layer. Afterwards, 436 nm LPL was irradiated across the entire area at 600 mJ cm^−2^ and 80 °C (Fig. 5b). Subsequently, 436 nm LPL orthogonal to that of the initial step was irradiated through a photomask under the same conditions (Fig. 5c).

Figure 5e,f display POM images of the resulting film under crossed polarizers rotated 45° from one another. As shown, the patterns containing the positive and negative contrast tones of the photomask were fully switched. This result clearly indicates successful planar-planar mode patterning of the mesogens in the PPBz film (scheme, Fig. 5d). This two-step photopatterning process demonstrates that the in-plane photoalignment can be readily overwritten by successive LPL irradiation. The overwriting could be repeated many times. A resolution of a few micrometres was obtained according to the discrimination of the patterns.

The command skin layer could be removed selectively with cyclohexane, as mentioned above (see Fig. 4b). Interestingly, the LC orientation patterning remained unchanged even after the removal of the photoresponsive surface skin layer. Consequently, a pure photopatterned PPBz film was obtained. (Supplementary Fig. 4) LPL irradiation at 80 °C was further performed to confirm the absence of photoalinment in this state. As expected, the film immediately turned to the homoeotropic state without in-plane preference (Supplementary Fig. 4d). Therefore, the photoalignment ability is fully lost after the removal of the skin layer.

### Homoeotropic-planar mode patterning by inkjet printing

**Figure 6:**
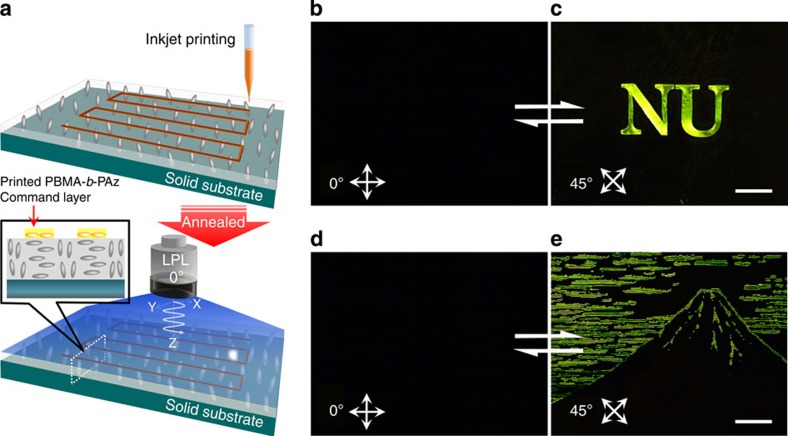
Homoeotropic-planar orientation mode patterning via inkjet printing. (**a**) Scheme of the inkjet printing on a PPBz thin film. After the inkjet printings were performed, the film was annealed at 125 °C for 10 min and successively irradiated with LPL at 80 °C. Panels **b** and **c** indicate the appearance and disappearance of a birefringence patterning of ‘NU’ characters in the POM observations under crossed polarizers 45° from one another. Panels **d** and **e** show another example of a drawn picture of a mountain. Note that the emergence of the images was fully switched during this procedure. Scale bar, 200 μm.

During the above patterning methods, the entire PPBz film area was covered with a photoresponsive top layer. Next, printing methods were used to place the layer at specific locations. Consequently, we chose an inkjet printing method, precluding any effects on the LC orientation caused by pressing during the printing process. PBMA-*b*-PAz was dissolved in *o*-dichlorobenzene (1% by weight) and applied using a subfemtolitre inkjet printer (scheme in Fig. 6a). With this apparatus, a printing width approaching 1 μm was available.

In the unprinted areas, the PPBz mesogens are oriented homoeotropically after annealing (see Fig. 3g), while in the printed regions, they adopt a planar orientation. Therefore, the patterning could occur between the homoeotropic and planar orientation modes (see Fig. 6a, inset cartoon). Only an annealing process without photoirradiation could make patterns in this mode (between Fig. 3g and h). In this case, the contrast of the POM images under crossed polarizers was not sufficient. The rotation of the crossed polarizers did not alter the bright/dark contrast, indicating that the bright regions were composed of randomly oriented planar domains.

When the photoalignment by LPL irradiation was also performed, refined clear patterns with dark/bright contrast were obtained during POM observation. In Fig. 6, two representative POM images of PPBz films obtained after inkjet printing PBMA-*b*-PAz, annealing and irradiation with LPL are displayed. Here, the letters ‘NU’ (Fig. 6b,c) and a drawing of a mountain (Fig. 6d,e) are shown. The printed images appeared and disappeared when rotating the crossed polarizers 45°. Therefore, the homogenous planar alignment (Fig. 3i) and the homoeotropic orientation (Fig. 3g) regions are patterned in the printed and unprinted parts, respectively. The resolution of this printing process was estimated using line patterns. A 1-μm line width was successfully obtained with some distortions. At a 5-μm width, a linear outline was clearly observed (Supplementary Fig. 5). We assume that a resolution of a few micrometres is appropriate for this process. Nearly identical resolutions are obtained for both patterning modes: planar-planar and homoeotropic-planar orientations.

These drawings may be performed by photopatterning with the corresponding photomask; however, the preparation of complex photomasks can be laborious and expensive. With the printing method, on-demand drawings containing any characters or figures are readily accessible, benefitting various applications.

### Variations in the substrate

The experiments were continued using various substrates. In addition to the clean quartz plate (*θ*_w_=10°–20°), the same procedures were performed over a hydrophobilized quartz surface (*θ*_w_=85°) treated with 1,1,1,3,3,3,-hexamethyldisilazane vapour. In addition, commercially available plastic sheets, such as polyimide and poly(ethylene terephthalate) films, were used as flexible, soft substrates. In all cases, the effective induction of birefringence and patterning on PPBz films could be performed in the same way, strongly suggesting that the nature of the substrate surface was not significant; the state of the free surface primarily controls the mesogen orientation in the film. With flexible polymer sheets, the substrates do not need to be flat, making any curved or fabricated substrates applicable.

## Discussion

The low molecular mass nematic LCs used in LC display panels are highly fluid and sandwiched between two solid substrate walls; therefore, the LC orientations are influenced only by the solid surfaces. In contrast, highly viscous or solidified LC materials, such as polymer LCs, are often exposed to air. Until now, the role of the free surface regarding the LC orientation has hardly been taken into account, except for the discotic LC film systems that form a hybrid orientation between an alignment film on a solid surface and the air^26^,^27^,^28^. In these cases, modifications on the solid substrate side are still required. In our approach, the solid surface side does not require any modification; manipulations on the free surface can control both in- and out-of-plane modes.

The optical alignment and patterning of side chain LC polymer films have been studied extensively for optical memory and photonic applications^29^,^30^,^31^,^32^,^33^. In these cases, the photoresponsive units are integrated with the LC polymer films. Therefore, the light must penetrate the films. However, by using a free surface command layer, the applicability of our method can be extended to various LC functional systems, including non-transparent LCs. For example, deeply coloured, light-emitting molecules or semiconducting materials exhibiting anisotropic functionalities may be embedded. Patterned polarized light emission devices and semiconducting circuits for transistors and solar cells are likely to be fabricated. Importantly, the surface command layer can be selectively removed while retaining the molecular orientation from the patterned LC film, allowing subsequent piling fabrications using another material. It is also necessary to consider the limitation of the present method. The supposed contamination of the surface-active polymer may deteriorate the properties of the LC film in the pure state. Further, fluid LC materials such as low molecular mass nematic LCs will not be applicable.

In both patterning procedures (photomask irradiation and inkjet printing), the molecular orientations of the film remain unchanged when room temperature is maintained. During inkjet printing, the high-contrast birefringent images appear only after annealing and LPL irradiation. Specifically, the free surface skin layer possesses latent images that are not recognized by optical detection or the naked eye during the initial stage. Plausible applications utilizing this property might include identification and protection against forgery.

In summary, the photoalignment processes proposed here are highly versatile in many aspects: no pretreatment of the substrate is required; various side chains LC polymer materials may be used (furthermore, the LC materials need not be transparent); simple annealing and LPL irradiation procedures are sufficient for developing clear images; on-demand patterns are readily available through a commercially available printing method; and various substrates, including inorganic and flexible polymer substrates, can be used. These simple but powerful methods should broaden the applicability of LC photoalignment to access various systems, providing new opportunities for optical^29^,^30^ and mechanical^31^,^34^ device fabrication.

## Methods

### Materials

4-Hydroxybenzoic acid, methacrylic acid were obtained from Aldrich. 6-Bromo-1-hexanol, hydroquinone, 4-pentyl phenol, 2,2′-azodiisobutyronitrile and 4-cyano-4′-hydroxybiphenyl were purchased from TCI. Unless otherwise stated, all other starting materials and reagents were purchased from commercial suppliers and used without further purification.

### Synthesis of polymers and diblock copolymer

The azobenzene-containing block copolymer (PBMA-*b*-PAz) was described in the supporting information of the previous paper^21^. The averaged numbers of repeating unit of PBMA and PAz used for this work were 510 and 144, respectively.

### Synthesis of 4-(6-hydroxyhexyloxy)benzoic acid

4-Hydroxybenzoic acid was dissolved in ethanol. Potassium hydroxide and catalytic amount of potassium iodide were added dropwise to the above solution. 6-Bromo-1-hexanol was then added dropwise under stirring. The solution was stirred for 3 h at 80 °C. Next, the solution was dissolved in methanol, acidified with hydrochloric acid and solvent was removed by filtration. The precipitate was recrystallized from hexane twice to give white platelet crystals. Yield: 7.8 g (24.0%).

### Synthesis of 4-(6-methacryloyloxyhexyloxy) benzoic acid

Methacrylic acid, 4-toluene sulphonic acid, hydroquinone and ethyl acetate were added to 4-(6-methacryloyloxyhexyloxy) benzoic acid in dry tetrahydrofuran (THF) solution, and then the mixture was stirred for 18 h at 80 °C. The reaction mixture was washed with water and hydroquinone was added. The solution was dried over anhydrous magnesium sulphate and then was purified using columun chromatography. This product was recrystallized from ethyl acetate to give white solid. Yield: 5.3 g (69.0%).

### Synthesis of phenyl benzoate monomer

Hydroquinone and thionyl chloride were added to 4-(6-methacryloyloxyhexyloxy)-4′-pentylphenyl benzoate, and then mixture was stirred for 2 h at room temperature under Ar gas. Diethylether was added dropwise under stirring until distilled thionyl chloride by azeotropy. After the solution was stirred at 110 °C, the product obtained was 4-(6-methacryloyloxyhexyloxy) benzoic chloride (BC6MA). Then, triethylamine was added to 4-pentyl phenol in dry THF solution. The mixture solution was added dropwise to BC6MA in dry THF solution and stirred for 2 h at room temperature. The reaction mixture was washed with water to neutralize and hydroquinone was added. The solution was dried over anhydrous magnesium sulphate and then was purified using columun chromatography. This product was recrystallized from ethyl acetate to give white solid. Yield: 2.4 g (56.0%).

### Polymerization of phenyl benzoate monomer

PPBz was synthesized by free radical polymerization in dry THF solution under nitrogen, using 2,2′-azodiisobutyronitrile as an initiator via free radical polymerization of the 4-(6-methacryloyloxyhexyloxy)-4′-pentylphenyl benzoate monomer. The reaction medium was heated at 60 °C for 24 h, cooled to room temperature and then poured into a vigorously stirred hexane for reprecipitation. The resulting polymer was collected by centrifugation. The white solid product was dried in vacuum. The reaction conversion was 95% (yield: 172 mg).

### Synthesis of 6-cyanobiphenoyl-1-hexanol

Potassium carbonate was added to 4-cyano-4′-hydroxybiphenyl in dry *N*,*N*-dimethylformamide solution under N_2_ gas. 6-Bromo-1-hexanol in dry *N*,*N*-dimethylformamide was then added dropwise under stirring. The solution was stirred for 2 h at room temperature. After this, the solution was dissolved in water, and extracted with chloroform and water. The precipitate was recrystallized from methanol twice to give white platelet crystals. Yield: 8.35 g (95.0%).

### Synthesis of 6-cyanobiphenyloxy-1-hexyl methacrylate

Trietylamine was added to 6-cyanobiphenoyl-1-hexanol in dry THF solution. A dry THF solution of methacryloyl chloride was added dropwise to the solution at 0 °C. The mixture was stirred for 30 min at 50 °C and 12 h at room temperature. The reaction mixture was washed with water. The solution was extracted with chloroform and water, and then dried over anhydrous magnesium sulphate. The solvent was evaporated and the residue was recrystallized from methanol. Yield: 5.4 g (60.0%).

### Polymerization of 6-cyanobiphenyloxy-1-hexyl methacrylate

CuCl, 4,4′-dinonyl-2,2′-dipyridyl and 6-cyanobiphenyloxy-1-hexyl methacrylate were added into a 15-ml pressure glass tube. The mixture was dissolved in THF in glove box. The resulting mixture was stirred for 10 min, and then EBB was added. The sealed mixture solution was removed from glove box and placed for 10 h in ChemStation at 70 °C. The polymerization was terminated by exposing the catalyst to air. The reaction solution was dissolved in chloroform and passed through an activated neutral alumina column to remove the Cu catalyst. After being concentrated, the solution was poured to hexane to remove CB6MA monomer. The reaction conversion was 55%.

### ^1^H NMR measurements

^1^HNMR spectra were recorded on a JNM-GSX270 (JEOL) in 16 steps using tetramethylsilane as the internal reference for deuterated chloroform solution (Across). A small amount of polymerized solution was measured and conversion of polymerization was calculated by value of integral of a monomer and a polymer.

### Gel permeation chromatography

GPC was performed useing a Shodex DS-4/UV-41/RI-101 and two GPC columns (Shodex KF-403 and KF-405). THF was used as an eluent at a flow rate of 1.0 ml min^−1^. The calibration of molecular weight was performed by using polystyrene standards (TSK standard polystyrene, TOSOH).

### Differential scanning calorimetry

DSC was performed with a Q200 (TA Instruments). DSC scans were performed within the temperature range 0–140 °C at a heating rate of 5.0 °C min^−1^ under nitrogen. About 5.0 mg mass was used for DSC measurements for all samples. An empty aluminium pan was used as a reference.

### Ultraviolet–visible absorption spectroscopy

Polarized ultraviolet–visible absorption spectra were taken on an Agilent 8453 spectrometer (Agilent Technologies) equipped with a polarizer in front of the sample. A D_2_-W lamp was used for illumination source. Qualitative evaluations of the mesogen orientation (homoeotropic or planar) were made by measuring absorption intensities at a wavelength of the π−π* band around 260, 300 and 350 nm for phenyl benzoate, cyanobiphenyl and azobenzene mesogens, respectively.

### X-ray measurements

GI-SAXS measurements were performed using a FR-E X-ray diffractometer and R-AXIS IV two-dimensional detector (Rigaku Co.). The scatterings were taken at 45 kV and 45 mA over 30 min using copper Cu Kα radiation (*λ*=1.542 Å), and the camera length was 300 mm. The two-dimensional X-ray scattering patterns were recorded on an imaging plate (Fujifilm Co.). The temperature of the film sample was controlled using a ceramic heater.

### Polarized optical microscope

The birefringent character of LC films were evaluated by optical microscope under crossed polarizers using a BX51 (Olympus Co.).

### Film preparation

The thin films of the homopolymer and blended polymer with 3 weight % PBMA-*b*-PAz were prepared by spincoating from chloroform solutions containing 2 weight % PCB or PPBz. Blended PBMA-*b*-PAz (3%)/PPBz cast films were prepared by casting from a 1 weight % chloroform solution. These films were annealed at 125 °C for 10 min. The evaluation of film thickness was made by atomic force microscopy using a Nanopics 2100 (Seiko Instruments).

### Contact angle measurements

The contact angle of a water droplet was estimated with a FACE CA-XP contact angle meter (Kyowa Interface Science). The averaged values of five measurements were obtained.

### Inkjet printing

The orientation patterning thin film was manufactured using a subfemtolitre inkjet printing apparatus and a drawing data system (SIJ Technology Co.). A PBMA-*b*-PAz solution in *o*-dichlorobenzene (1 weight %) was injected from a drawing nozzle under generated voltage. Lines were drawn at a voltage of 600 V and frequency of 60 Hz, with a distance between the substrate and the nozzle of 30 μm. Printed images of characters of ‘NU’ and a mountain were drawn with a computer control system using a DXF-SIJ software. After printing, the film was annealed at 125 °C for 10 min and successively irradiated with LPL (600 mJ cm^−2^).

### Light irradiation

LPL irradiation was performed with a REX-250 (Asahi Spectra Co.) equipped with a polarizer. The films were irradiated with LPL at 436 nm (visible) and 600 mJ cm^−2^. The PPBz and the blended PBMA-*b*-PAz (3%)/PPBz thin films were irradiated with 436 nm LPL at 80 °C, while the PCB and the blended PBMA-*b*-PAz (3%)/PCB thin films were irradiated at 100 °C. These temperatures were chosen as to be set slightly below the isotropidisation temperatures of PPBz or PCB.

## Author contributions

T.S. and S.N. conceived the project and designed the experiments. K.F. contributed to the polymer synthesis, the experimental procedures and the measurements. M.H. contributed assistance in performing procedures, especially X-ray analysis. All members analysed and discussed the results. T.S. and K.F. co-wrote the manuscript.

## Additional information

**How to cite this article:** Fukuhara, K. *et al.* Free-surface molecular command systems for photoalignment of liquid crystalline materials. *Nat. Commun.* 5:3320 doi: 10.1038/ncomms4320 (2014).

## Supplementary Material

Supplementary Information Supplementary Figures 1-5, Supplementary Note 1 and Supplementary Reference

## Figures and Tables

**Figure 1:**
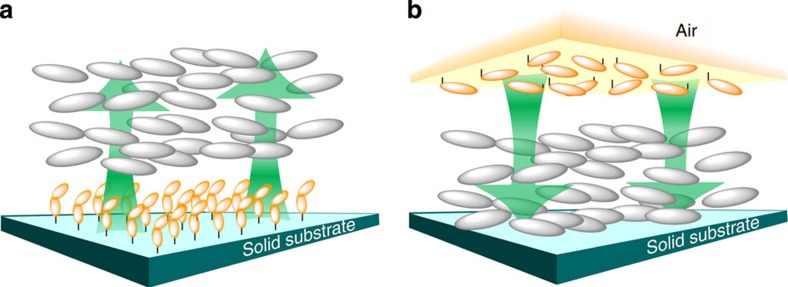
Schematic illustrations of surface photoalignment controls. (**a**) Alignment control of LC molecules using surface photoreactions from the solid substrate side (command surface effect). (**b**) New proposal to exert a command effect with a photoresponsive skin layer existing on the free (air) surface side.
