# Supplementary material for: Free-surface molecular command systems for photoalignment of liquid crystalline materials
Source: Nat Commun. 2014 Feb 18;5:3320. doi: 10.1038/ncomms4320 (PMC4355354; doi:10.1038/ncomms4320)
Supplement: Supplementary Information — Supplementary Figures 1-5, Supplementary Note 1 and Supplementary Reference [file ncomms4320-s1.pdf]

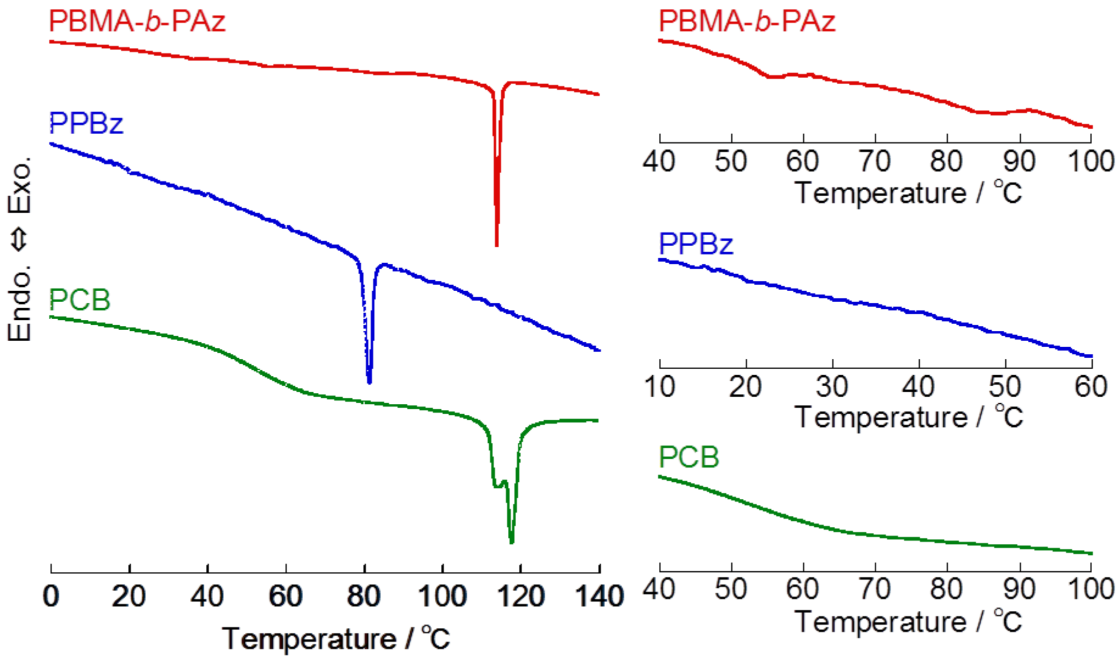

Supplementary Figure 1. DSC curves of the block copolymer and homopolymer on the second heating in ranges from 0 to 140 °C. The heating rate is 5.0 °C min^{-1}. Curves for PBMA-*b*-PAz, PPBz and PCB are indicated.

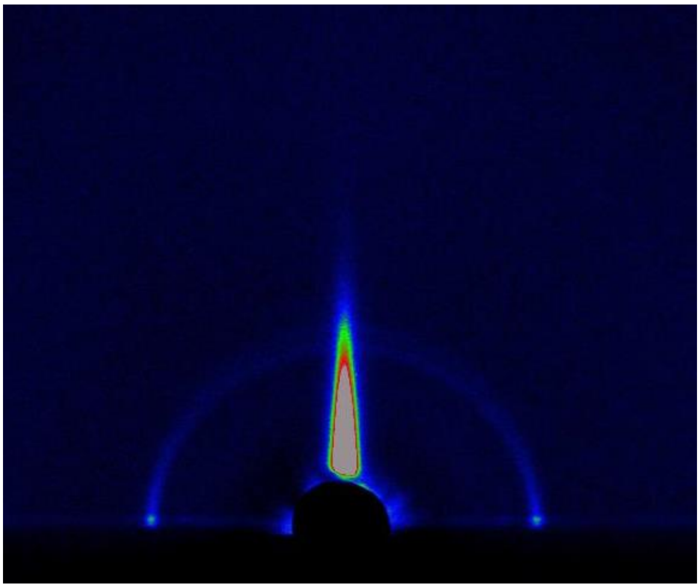

Supplementary Figure 2. The 2D GI-XRD patterns shows the PBMA-*b*-PAz (3 %)/PPBz blend thin film before annealing.

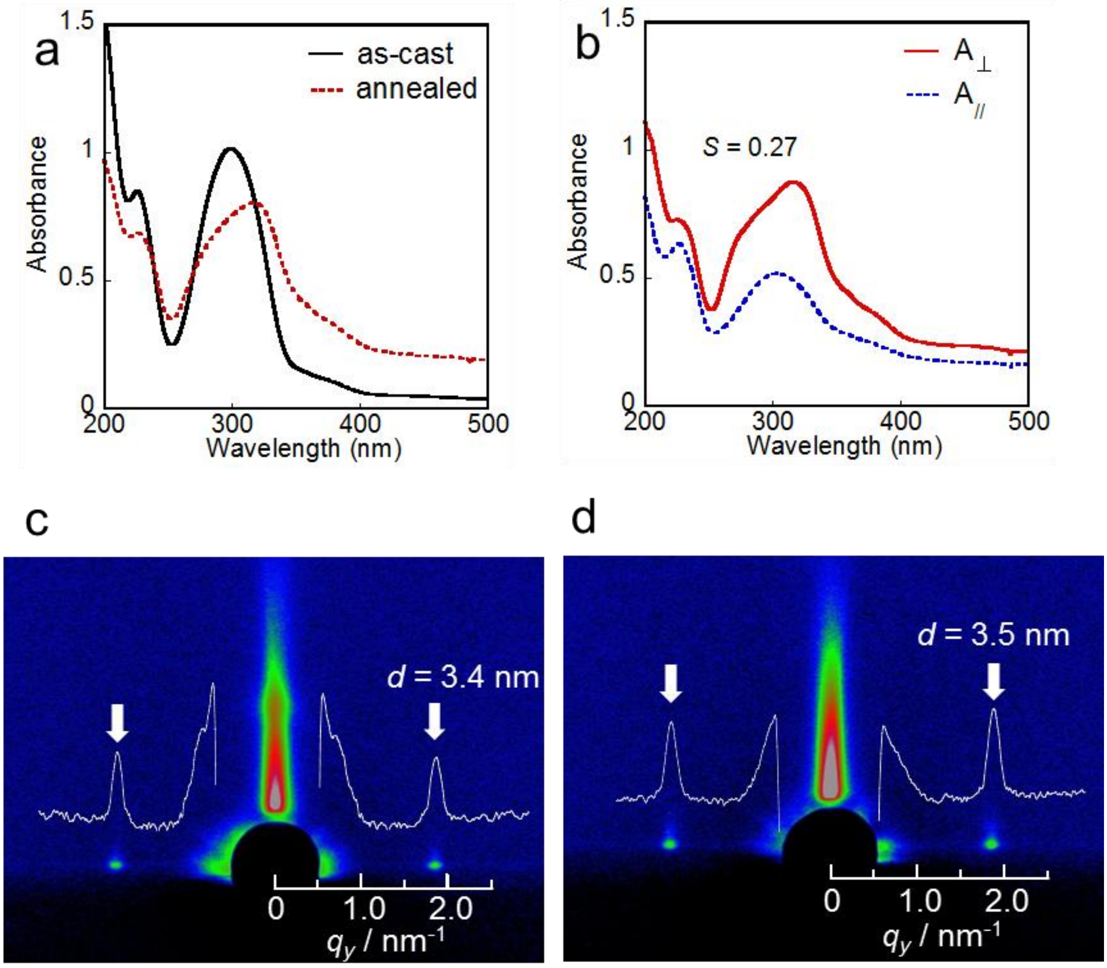

Supplementary Figure 3. UV-Vis absorption spectra of the PBMA-*b*-PAz (3 %)/PCB blend thin film (solid line) and after annealing at 125 °C (dotted line) (**a**) and after irradiation with 436 nm LPL at 1000 mJ cm^{-2} (**b**), respectively. The spectra were taken with the probing beam parallel (dotted line) and orthogonal (solid line) to the actuating light. The 2D GI-XRD patterns shows the PBMA-*b*-PAz (3 %)/PCB blend thin film after annealing at 125 °C (**c**) and LPL-irradiated (**d**), respectively (X-ray incidence: orthogonal to the actinic LPL). In the XRD patterns, 1D intensity profiles are indicated as white lines.

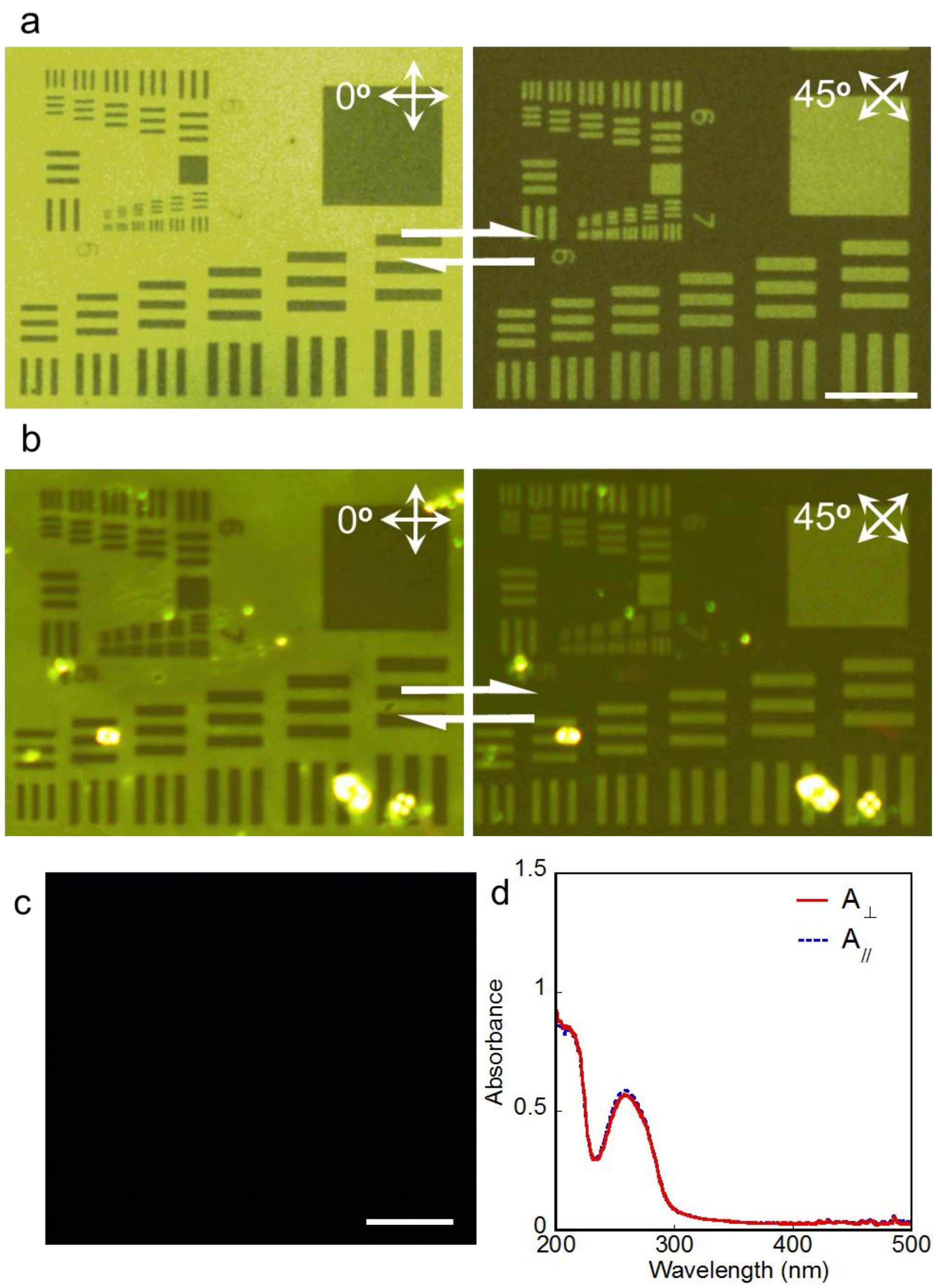

Supplementary Figure 4. The POM images of the PBMA-*b*-PAz (3 %)/PPBz blend thin film after irradiating with LPL used by the photomask (**a**), after rinsing in cyclohexane for 5 min (**b**) and after irradiating with LPL. These were observed by difference contrasts at rotating 0 and 45 °. POM image (**c**) and UV-Vis absorption spectra of the rinsed film after successive irradiation with 436 nm LPL at 10 mJ cm^{-2} are also displayed. In **d**, the spectra were taken with the probing beam parallel (dotted line) and orthogonal (solid line) to the actinic light. Scale bar (**a, b** and **c**): 100 μm.

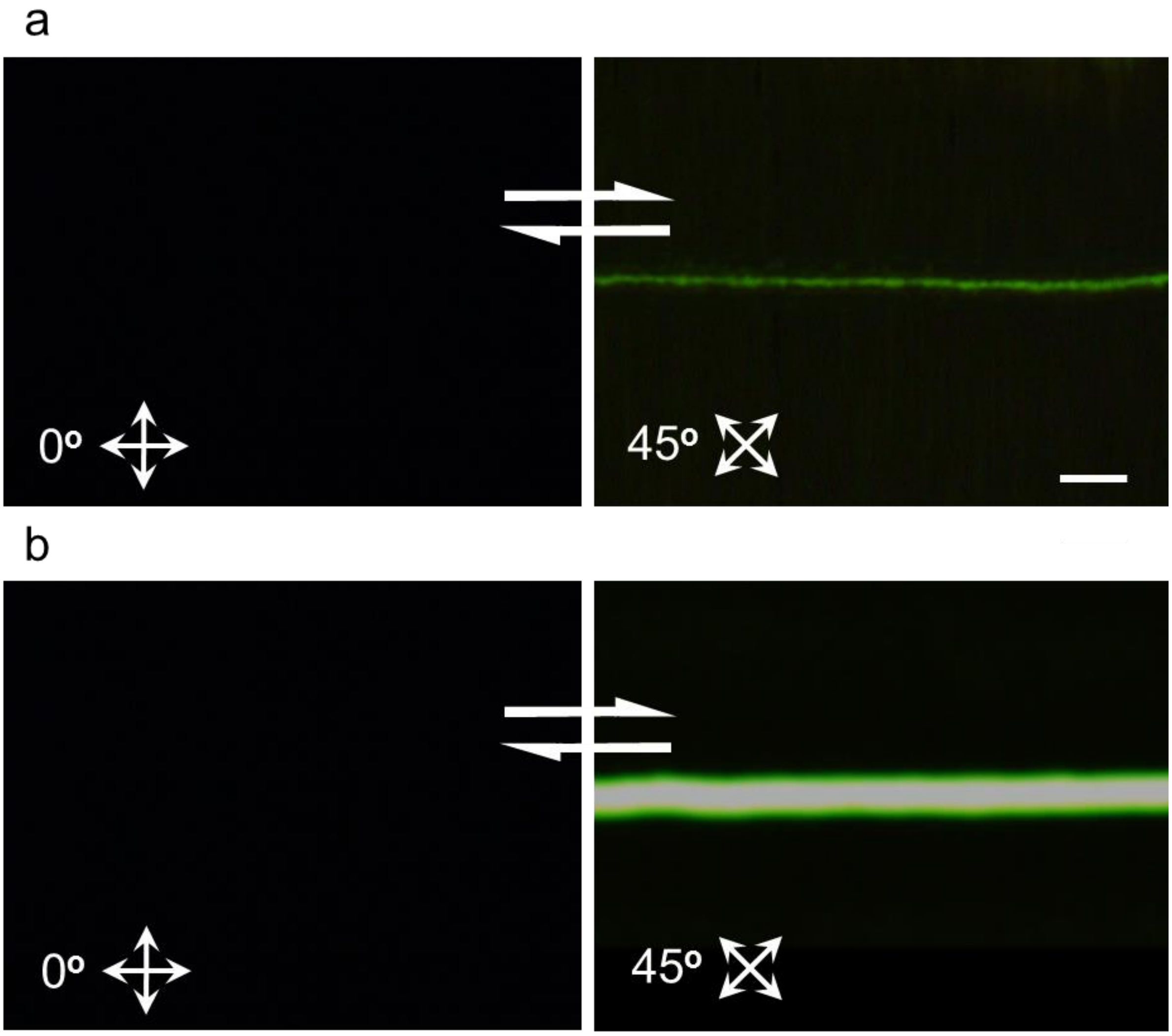

Supplementary Figure 5. The POM image of the PPBz film after inkjet printing drawings of PBMA-*b*-PAz and successive annealing (125 °C for 10 min) and irradiation with LPL (600 mJ cm^{-2}). These was observed by diffrence contrasts at rotating 0 and 45°.Scale bar: 10 μm.

Note 1: On the photoinduced ordering

The in-plane orientational order parameter (S) was estimated by $S = (A_{\perp}-A_{||})/(A_{\perp}+2A_{||})$, where $A_{\perp}$ and $A_{||}$ denote the absorbance at the peak maximum obtained through measurements using polarised light with E perpendicular and parallel to that of the actinic polarised light, respectively. Note that the $A_{\perp}$ and $A_{||}$ in this paper are defined for the direction of actinic polarised light, and not for the director of LC molecule which is usually used for the determination by spectroscopic measurements.[1] Based on the definition of $A_{\perp}$ and $A_{||}$ by the LC director, the $S = (A_{\perp}- A_{||})/(A_{\perp}+2A_{||})$ will give minus in sign. However, by the definition in this paper based on the direction of actinic LPL, this calculation gives positive S values in sign.

Supplementary Reference

1 D. Dummur and K. Toriyama, Optical Properties in *Handbook of Liquid Crystals* (D. Demus, J. Goodby, G. W. Gray, H.-W. Spiess eds), Vol. 1, pp. 215-230, Wiley-VCH, Weinheim (1998)).
